# Mixed Triboelectric and Flexoelectric Charge Transfer at the Nanoscale

**DOI:** 10.1002/advs.202101793

**Published:** 2021-08-13

**Authors:** Huimin Qiao, Pin Zhao, Owoong Kwon, Ahrum Sohn, Fangping Zhuo, Dong‐Min Lee, Changhyo Sun, Daehee Seol, Daesu Lee, Sang‐Woo Kim, Yunseok Kim

**Affiliations:** ^1^ School of Advanced Materials and Engineering Sungkyunkwan University (SKKU) Suwon 16419 Republic of Korea; ^2^ Research Center for Advanced Materials Technology Sungkyunkwan University (SKKU) Suwon 16419 Republic of Korea; ^3^ Department of Materials and Earth Sciences Technical University of Darmstadt 64287 Darmstadt Germany; ^4^ Department of Physics Pohang University of Science & Technology (POSTECH) Pohang 37673 Republic of Korea; ^5^ SKKU Advanced Institute of Nanotechnology (SAINT) and Samsung Advanced Institute for Health Sciences & Technology (SAIHST) Sungkyunkwan University (SKKU) Suwon 16419 Republic of Korea

**Keywords:** atomic force microscopy, charge transfer, flexoelectricity, triboelectricity

## Abstract

The triboelectric effect is a ubiquitous phenomenon in which the surfaces of two materials are easily charged during the contact‐separation process. Despite the widespread consequences and applications, the charging mechanisms are not sufficiently understood. Here, the authors report that, in the presence of a strain gradient, the charge transfer is a result of competition between flexoelectricity and triboelectricity, which could enhance charge transfer during triboelectric measurements when the charge transfers of both effects are in the same direction. When they are in the opposite directions, the direction and amount of charge transfer could be modulated by the competition between flexoelectric and triboelectric effects, which leads to a distinctive phenomenon, that is, the charge transfer is reversed with varying forces. The subsequent results on the electrical power output signals from the triboelectrification support the proposed mechanism. Therefore, the present study emphasizes the key role of the flexoelectric effect through experimental approaches, and suggests that both the amount and direction of charge transfer can be modulated by manipulating the mixed triboelectric and flexoelectric effects. This finding may provide important information on the triboelectric effect and can be further extended to serve as a guideline for material selection during a nanopatterned device design.

## Graphical abstract

Using experimental approaches, the authors show that, in the presence of strain gradient, the charge transfer during triboelectric measurements is resulted from competition between flexoelectricity and triboelectricity. The charge transfer can be enhanced or inversed depending on the relative charge transfer direction of both effects. This finding could be a guideline for material selection for a nanopatterned device design.

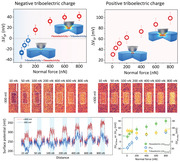

## 1 Introduction

Triboelectricity is a process by which material surfaces become electrically charged as a result of touching or rubbing another surface.^[^ ^1^, ^2^, ^3^ ^]^ This effect is well known and has widespread and significant influences. Based on a charge transfer, triboelectricity has garnered wide attention because of its great potential in applications in various areas such as electrostatic self‐assembly,^[^ ^4^ ^]^ ionic electret,^[^ ^5^ ^]^ and triboelectric nanogenerators,^[^ ^6^, ^7^ ^]^ which have been widely studied for applications in various fields.^[^ ^8^, ^9^, ^10^ ^]^ Despite its elementary nature and high application value, the fundamental mechanism of triboelectricity has not been fully understood,^[^ ^1^ ^]^ particularly the involvement of dielectrics and the reduction of size to the micro/nanoscale, which are common in state‐of‐the‐art nanotechnologies, make the question even more complicated.

In contrast, flexoelectricity, the coupling between an electric polarization and strain gradient,^[^ ^11^, ^12^, ^13^, ^14^, ^15^ ^]^ has been demonstrated in recent studies on various material systems to modulate physical properties, for example, a photovoltaic effect, Schottky barrier, resistance, or polarization, has been reported in BiFeO_3_,^[^ ^16^ ^]^ halide perovskite,^[^ ^17^ ^]^ Si,^[^ ^18^ ^]^ TiO_2_,^[^ ^18^, ^19^ ^]^ and (Nb‐)SrTiO_3_,^[^ ^14^, ^15^, ^18^, ^19^ ^]^ to name a few. Basically, flexoelectricity is a universal property of all dielectric materials including centrosymmetric materials, which occurs under an inhomogeneous strain field.^[^ ^14^, ^17^ ^]^ Although it is negligible at the bulk scale level owing to a small strain gradient,^[^ ^20^ ^]^ it cannot be ignored at the nanoscale level because the strain gradient at this level is several times larger than at the bulk scale level.^[^ ^12^ ^]^ Therefore, when a dielectric material is subjected to an inhomogeneous force at the nanoscale, the strain gradient breaks the original symmetry and forms a polarization with a preferred direction.^[^ ^19^ ^]^ It has been reported the polarization in ferroelectrics can modulate the triboelectric charge transfer,^[^ ^21^ ^]^ likewise, the flexoelectric effect in centrosymmetric dielectrics may also contribute to the charge transfer in the presence of a strain gradient. Recently, theoretical analyses suggested that bipolar current in the triboelectric devices were originated from the surface potential difference induced by flexoelectricity, that is, the triboelectricity is a result of flexoelectricity.^[^ ^22^ ^]^ Based on conventional Herzian and Johnson–Kendall–Roberts contact models, the authors studied surface potential difference by flexoelectricity in indentation and pull‐off cases and suggested flexoelectricity was a thermodynamic driver in triboelectric phenomena. Indeed, following studies by similar method and density functional theory suggested it could be used to theoretically explain the charge transfer between identical materials, where the work function difference is absence.^[^ ^23^, ^24^ ^]^ Nevertheless, it cannot provide a satisfactory interpretation on many reports on charge transfer between different materials, which are readily explained by work function difference.^[^ ^3^ ^]^ On the other hand, direct experimental evidence on the role of flexoelectricity on the charge transfer between different materials, that is, in the presence of work function difference, is scarce to date. Therefore, more efforts are needed to show the direct evidence and have a clear understanding of the contribution of flexoelectricity to triboelectric charge transfer, especially between different materials.

The condition described above is easily achievable in an atomic force microscopy (AFM)‐based measurement,^[^ ^14^, ^25^, ^26^, ^27^ ^]^ which is also a general approach to settle the fundamental questions of the triboelectric effect at the nanoscale.^[^ ^2^, ^21^, ^28^, ^29^ ^]^ The triboelectric charge transfer is usually realized by rubbing the sample surface with a sharp AFM tip under various conditions.^[^ ^30^, ^31^ ^]^ During this process, an inhomogeneous force is exerted on the surface, inducing a strain gradient in the sample beneath the tip; thus, the flexoelectric effect can play a role in the charge transfer when rubbing. Because of the high sensitivity to the strain gradient, and thus force, the flexoelectric effect might be distinguishable by varying the force exerted on the surface while keeping the other conditions unchanged. Nevertheless, to the best of our knowledge, detailed force‐dependent triboelectric charge transfer at nanoscale has yet to be experimentally reported. Furthermore, making this question clear is not only of significance to the fundamental scientific understanding of triboelectric charging, but also important for the device design and applications. For instance, it was observed that the electrical power output from the triboelectrification can be significantly modified by fabricating nano‐ and micro‐scale structures on one of the surfaces.^[^ ^32^, ^33^, ^34^, ^35^, ^36^ ^]^ In these cases, although the variation in the contact area has been employed to explain the observations, the flexoelectric effect might also play a role in modulating the power output because the flexoelectric effect becomes non‐negligible at the nanoscale owing to the gigantic strain gradient.^[^ ^25^ ^]^ Consequently, experimentally confirming how the flexoelectric effect influences the charge transfer during triboelectric measurement is an important aspect of the triboelectric mechanism.

Herein, we propose that the charge transfer between a sharp metal and flat oxide thin film can be attributed to mixed triboelectric and flexoelectric effects instead of pure triboelectric or flexoelectric effects, and the enhancement of charge transfer could be achieved when the charge transfers of both effects are in the same direction. To demonstrate this, force‐dependent triboelectricity was explored using AFM. Contact mode AFM was used to rub the sample surface using an AFM tip with a controllable normal force. Subsequently, the triboelectric charge distribution and subsequent diffusion on the surface were characterized using Kelvin probe force microscopy (KPFM).^[^ ^37^ ^]^ Because the surface potential is determined by the work function difference between the AFM tip and sample surface,^[^ ^38^ ^]^ the variation of the work function or charge transfer can be analyzed through surface potential images. It turns out that, in the case of opposite triboelectric and flexoelectric charges, the dominant contributor alters from the triboelectricity in the low‐force regime to flexoelectricity in the high‐force regime. Furthermore, triboelectric charging can be improved by flexoelectricity when they are in the same direction. The subsequent results obtained by applying a small positive/negative tip bias further validate this concept. In addition, the electrical power outputs from triboelectrification between two flat surfaces and flat‐nano pattern pairs further confirm the validity of the proposed mechanism. The present study suggests that triboelectricity is inevitably coupled with flexoelectricity in nanoscale systems where a strain gradient is generated. We demonstrated an experimental pathway to visualize the contribution of flexoelectricity to triboelectricity, which sheds light on the understanding of triboelectric charges at the nanoscale and provides a guideline for the design of high‐performance nanoscale triboelectric devices by selecting suitable materials.

## 2 Results and Discussion

To explore how the flexoelectricity contributes to the triboelectric measurements, we chose a TiO_2_ thin film, of which the physical parameters relevant to flexoelectricity have been reported in various studies,^[^ ^18^, ^19^ ^]^ and a conductive diamond‐coated tip (CDT‐FMR), the high hardness of which can minimize the tip wear during measurements, as a model system. It has been suggested that a downward flexoelectric polarization associated with the strain gradient could be generated beneath the tip when an AFM tip is pressed against the film surface, which resembles the case of a positive voltage applied to the tip in the case of TiO_2_.^[^ ^26^, ^39^, ^40^, ^41^, ^42^, ^43^ ^]^ Therefore, the flexoelectricity is supposed to favor electron transfer from the sample to the tip. In this case, an opposite triboelectric charge transfer, that is, from the tip to the sample, would be ideal to study the coupling between the two, which requires a higher work function for the sample than the tip. The calibrated work functions of the tip and sample are *φ*_tip,eff_ = 4.52 eV and *φ*_sample,eff_ = 4.8 eV, respectively (see the details in Figure S1, Supporting Information). These characteristics make them an optical platform for our purposes.

**Figure 1:**
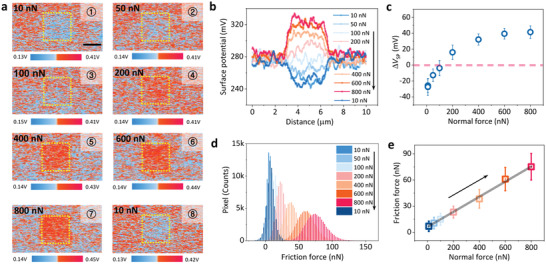
a) Surface potential images after rubbing with different normal forces on TiO_2_ thin film. Scale bar is 2 µm. b) Corresponding surface potential profiles, c) Δ*V*_SP_ between rubbed and pristine regions, d) histograms of friction force at different normal forces, and e) friction force as function of normal force corresponding to (d). Solid line in (e) is fitted using linear function. Numbers in (a) and black arrows in (b,d,e) indicate measurement sequence. For all data, scan rate is 0.5 Hz with CDT‐FMR.

The occurrence of triboelectricity only requires physical contact, whereas flexoelectricity is linearly dependent on the strain gradient, which is sensitive to the normal force. Therefore, we examined the charge transfer dependence on the normal force applied by the AFM tip during rubbing. The amount and direction of transferred charges between the tip and sample can be evaluated by the change in the surface potential between the rubbed and pristine areas. **Figure** **1**a displays the surface potential distribution in the areas rubbed with normal forces ranging from 10 to 800 nn. After being rubbed by a low normal force (<50 nn), the area is relatively negatively charged because it has a lower surface potential than the surrounding pristine area. In contrast, a high normal force (>200 nn) induces a higher surface potential than the surrounding pristine area, indicating that it is relatively positively charged. This normal force‐dependent surface potential variation is more clearly visible in both surface potential line profiles (Figure 1b) and surface potential difference (Δ*V*_SP_) between the rubbed (*V*_SP,r_) and surrounding pristine (*V*_SP,p_) areas (Δ*V*_SP_ = *V*_SP,r_ − *V*_SP,p_) as a function of the normal force (Figure 1c). The sign of Δ*V*_SP_ changes nonlinearly from negative to positive across zero as the normal force increases.

During surface rubbing, the friction signal, or lateral signal (in volts) distribution at each normal force, was simultaneously recorded. Through a friction force calibration using the modified Wedge–Flat method,^[^ ^44^, ^45^ ^]^ the friction force (in nanonewtons) was obtained from the friction signal (see the details in Section S1 and Figure S2, Supporting Information). The histograms of the friction force depending on the normal force and the relationship between the normal and friction forces are shown in Figures 1d and 1e, respectively. It can be seen that the friction force increases linearly with the normal force, as expected. Therefore, we can infer that, because the friction force increases linearly with an increasing normal force, a higher normal force can induce a greater charge transfer associated with the triboelectric effect.

**Figure 2:**
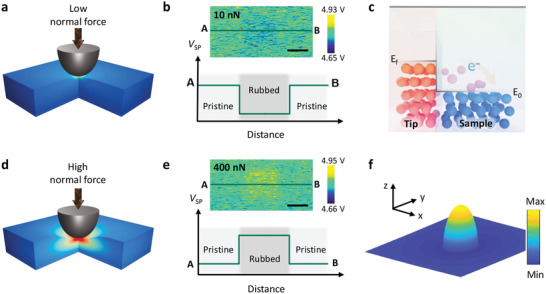
a,d) Schematics of tip‐sample contact under a) low and d) high normal force and b,e) corresponding (top) representative work function images and (bottom) illustrative surface potential profile across rubbed region under b) low and e) high normal forces. Scale bar is 2 µm. c) Triboelectric charge transfer driven by work function difference where *E*_f_ and *E*_0_ are Fermi level of tip and highest occupied surface state level of sample, respectively. f) Simulated flexoelectric surface potential on TiO_2_ thin film at normal force of 500 nn, that is, high normal force, from tip.

First, we attempted to use triboelectricity to explain the intriguing charge transfer results without considering the flexoelectricity. The schematics in **Figure** **2**a–c shows the charge flow driven by the work function: when the tip and sample are physically in a contact state, electrons flow from one to another driven by the difference in work function (see Figure 2c).^[^ ^46^, ^47^ ^]^ According to the calibrated work functions, the Fermi level (*E*_f_) of the tip is higher than the highest occupied surface state level of the sample. Therefore, the electrons in the tip will migrate to the sample surface to fill up the surface energy states at the same height as *E*_f_ of the tip when they are in contact,^[^ ^48^ ^]^ as shown in Figure 2c. Consequently, a lower surface potential is observed in the rubbed area compared with the pristine state as an indicator of a lower work function, as depicted in Figure 2b, confirming the dominant role of the triboelectricity at a low normal force. When the normal force increases, the triboelectric charge transfer is considered to increase because of the increased friction force.^[^ ^49^, ^50^ ^]^ Meanwhile, the contact area will increase until reaching an extreme state,^[^ ^51^ ^]^ which has been reported to promote the triboelectricity.^[^ ^52^ ^]^ If triboelectricity is the only contribution, as generally considered, the triboelectric charge should have a positive correlation with the normal force, as well as the friction force. In other words, more triboelectric charges would transfer from the tip to the sample at a higher normal/friction force, and the charge flow direction would not be altered by the force. Thus, we can rule out the influence of increasing contact area on the observed sign reversal result. Consequently, the triboelectric effect alone provides an unsatisfactory interpretation of the sign reversal phenomenon shown in Figure 1.

Alternatively, when considering the coupling of flexoelectricity with triboelectricity, we can find a plausible explanation for these fascinating observations. In the case of an extremely low normal force, the flexoelectric effect is assumed to be negligible because of the small strain gradient. With increasing normal force, the strain gradient in the TiO_2_ thin film gradually increases to a degree that cannot be ignored, and the flexoelectricity becomes involved as a competitor against the triboelectricity. The schematics in Figure 2d,e illustrates the strain gradient and charge transfer driven by the flexoelectricity at a high normal force, which is in the opposite direction compared to that of triboelectricity, that is, from sample to tip, in the current system. Accordingly, when the flexoelectricity charge is dominant over the triboelectric charge, the rubbed area exhibits a higher surface potential. To confirm the flexoelectric contribution, we use contact mechanics analysis to simulate the strain gradient, flexoelectric field, which results from flexoelectric polarization, and the corresponding electric potential in the TiO_2_ thin film under an AFM tip with relatively low and high normal forces.^[^ ^53^ ^]^ The details can be found in Section 3, Supporting Information. As expected, the strength and coverage of the strain gradient and flexoelectric field were pronounced at higher normal forces, as shown in Figure S3, Supporting Information. Furthermore, we were able to obtain the electric potential on the surface associated with flexoelectricity, that is, the flexoelectric surface potential (*V*_flexo_), as demonstrated in Figure 2f and Figure S3, Supporting Information. Indeed, the simulated flexoelectric surface potential is relatively higher than that of the surrounding area, as shown in Figure 2e. Based on the calculation, the dependence of *V*_flexo_ on the normal force can be determined theoretically (Figure S4b, Supporting Information). It is worth noting that the *V*_flexo_ used here is the maximum value of the flexoelectric surface potential under a certain condition,^[^ ^22^ ^]^which could differ from the actual value in the experiment. Furthermore, the Schottky barrier can be modulated through flexoelectric polarization, which could be another contributor to triboelectric charging.^[^ ^18^ ^]^

The observed charge transfer in our case is in nature a result of mixed triboelectric and flexoelectric effects, particularly at high normal forces. The triboelectric and flexoelectric effects generate negative and positive charges, respectively, on the TiO_2_ thin‐film surface. Therefore, the resultant charge transfer depends on the competition between the triboelectric and flexoelectric effects. At a low normal force, because the triboelectric effect is dominant owing to the relatively weak flexoelectric effect, the rubbed area is negatively charged and shows a lower surface potential. When the normal force increases, the flexoelectricity gradually surpasses the triboelectricity and thus results in a charge sign that changes from negative to positive, as shown in Figure 1. According to our calculations and reported studies, the flexoelectric effect exhibits an exponential correlation with the force.^[^ ^54^, ^55^ ^]^ Note that, because the amount of change in Figure 1 is not significantly high, the saturated‐like behavior at a high normal force might not be relevant to a Coulombic repulsion.^[^ ^56^, ^57^, ^58^ ^]^ Furthermore, the ionic motion can be excluded as a main contribution owing to the reversibility. It is known that the triboelectric effect could generate heat and raised the temperature at the contact interface, which would also affect the charge transfer behavior by accelerating charge dissipation in the contextual situation.^[^ ^59^ ^]^ However, it cannot explain the change of charge transfer direction and increase positive surface potential at high normal force. Besides, the temperature change is not supposed to be high considering the small contact radius and pressure by comparing with references.^[^ ^60^, ^61^ ^]^ Overall, the influence of raised temperature by triboelectricity is not supposed to have a significant influence on the charge transfer observed in this work.

**Figure 3:**
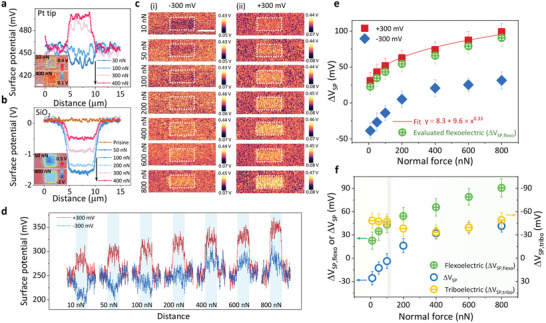
a,b) Surface potential profiles after rubbing with different normal forces in systems of a) Pt‐coated tip and TiO_2_ thin film, and b) conductive diamond‐coated tip and SiO_2_ thin film. Black arrows indicate the measurement sequence. Insets are representative surface potential images. Details are revealed in Figures S5 and S6, Supporting Information. Scale bar in inset is 3 µm. c) Surface potential images after rubbing with tip bias of i) −300 and ii) +300 mV at different normal forces on TiO_2_ thin film with conductive diamond‐coated tip and d) corresponding surface potential profiles. e) Normal force‐dependent Δ*V*_SP_ obtained from Figure 3d and evaluated flexoelectric contribution. f) Δ*V*_SP_ as a function of normal force without tip bias as in Figure 1c, Δ*V*_SP,flexo_ as in Figure 3e, and evaluated triboelectric contribution (Δ*V*_SP,tribo_ = Δ*V*_SP_ – Δ*V*_SP,flexo_).

To examine the universality of the mechanism, we conducted experiments in different systems with an alternative cantilever and sample, respectively. As shown in **Figure** **3**a,b and Figures S5 and S6, Supporting Information, a similar tendency of decreasing charge transfer with increasing normal force was observed when we changed the cantilever (Multi75E‐G, Pt‐coated) or sample (SiO_2_ thin film). Thus, we concluded that this mechanism may be universally valid in other systems, rather than merely valid for specific tips and samples. Meanwhile, we should note that because the triboelectricity is strongly dependent on the surface state of the sample or environmental conditions, that is, humidity, surface defects, and adsorbates, these additional factors could have a tremendous influence on the triboelectric charging behavior.^[^ ^62^, ^63^ ^]^ In addition, the variation of the local *I*–*V* curve with normal force, as shown in Figure S7, Supporting Information, also suggests a contribution from the flexoelectricity, the details of which can be found in the Supporting Information.

According to the concept proposed above, the charge transfer in question is the result of mixed triboelectric and flexoelectric effects instead of the triboelectric or flexoelectric effect alone. Thus, the flexoelectric effect is considered to enhance the triboelectric charge transfer when in the same direction, that is, a positive surface potential difference will be observed at a low normal force and increases with normal force. To avoid the possible uncertainties relevant to the physical properties, for example, flexoelectric coefficient, surface state, and defects, to name a few, examining the idea in the same system is ideal. The application of positive or negative bias to the tip can nullify the work function difference between the tip and sample, which resembles the basic operational principle of contact KPFM.^[^ ^64^ ^]^ Thus, if one can nullify the work function difference by applying a bias to the tip while rubbing, the triboelectric charge transfer driven by the work function difference can be minimized or even cause a reverse charge transfer owing to the flexoelectric effect. In contrast, a triboelectric charge transfer can be enhanced if an opposite sign of the bias is applied to the tip. Figure 3c(i) shows the surface potential images after rubbing the TiO_2_ film surface with different normal forces while applying a tip bias of −300 mV, which is opposite the nullifying bias. It shows a similar dependence on the normal force as in the case of zero tip bias (Figure 1), whereas the triboelectric charge transfer is enhanced and the critical normal force, at which no charge transfer is observed, increases. In contrast, the application of +300 mV, which is similar to (but slightly higher than) the work function difference between the tip and sample in Figure 1, nearly nullifies the original work function difference and leads to an opposite charge transfer direction. In addition, the charge transfer was enhanced with the normal force, as expected.

The surface potential line profiles in Figure 3d and the calculated Δ*V*_SP_ in Figure 3e present a distinct contrast in the surface potential after rubbing with a tip bias at various normal forces. With a slight discrepancy, the tip bias of +300 mV nullifies the work function difference between the tip and sample; therefore, Δ*V*_SP_ (+300 mV) is considered to be mainly contributed to by the flexoelectricity. We fitted the normal force‐dependent Δ*V*_SP_ (+300 mV) by using the exponential relationship as in the theoretical analysis and introduced an offset considering the discrepancy. Thus, the flexoelectric charge transfer (Δ*V*_SP,flexo_) can be evaluated by subtracting the offset from the experimental Δ*V*_SP_ (+300 mV), as demonstrated in Figure 3e. By assuming that Δ*V*_SP_ obtained in the experiment only contains triboelectric and flexoelectric contributions, it can be expressed as Δ*V*_SP_ = Δ*V*_SP,tribo_ + Δ*V*_SP,flexo_, where Δ*V*_SP,tribo_ indicates the triboelectric contribution to the experimental results. Therefore, Δ*V*_SP,tribo_ can be roughly estimated by subtracting Δ*V*_SP,flexo_ (Figure 3e) from Δ*V*_SP_ (Figure 1c), as shown in Figure 3f. The different tendency at low and high normal force regions could be a result of band bending induced through flexoelectric polarization and an increased contact area,^[^ ^65^ ^]^ suggesting a more complicated coupling between the triboelectric and flexoelectric effects rather than simply mixing. In Figure 3f, there is a crossover between the triboelectric and flexoelectric effects, where a near‐zero charge transfer is observed in Figure 1c, as marked by a gray shadow. It is noteworthy that the crossover of the flexoelectricity and triboelectricity, and thus the resultant normal force‐dependent charge transfer tendency, depend on various factors, such as the environmental condition, the work function difference between the tip and sample, the radius and Poisson's ratio of the tip, and the flexoelectric coefficient of the sample. Therefore, a crossover can be not observable in some circumstances. In our work, a sharp metal and flat oxide thin films were utilized considering it is a common system in AFM‐based triboelectric studies. Nevertheless, the sharp metal can be replaced by any kind of material as long as it can exert a non‐uniform force on the material it contacts to generate a large strain gradient. We note that, when the sharp metal is replaced by a dielectric material, the flexoelectricity in that material should be also considered. For samples, the analysis approach of flexoelectricity in this work could be applicable to most crystalline dielectric materials such as TiO_2_ and SiO_2_ shown here, noting physical properties relevant to flexoelectricity are disparate in different materials, for example, materials with large dielectric permittivity usually exhibit large flexoelectricity.^[^ ^13^ ^]^ Meanwhile, the redistribution of defects should be also taken into consideration when the dielectric materials are doped with high concentration of defects, for example, oxygen vacancies. If the dielectric thin film itself is not flat (e.g., corrugation surface) and exerted by a non‐uniform force, there will be intrinsic flexoelectric polarization by corrugation surface,^[^ ^40^ ^]^ which would also contribute to the resultant charge transfer. In the case of dielectric bulks, flexoelectricity is generally supposed to be negligible because of relatively small strain gradient, while in certain cases, for example, bending a flake, flexoelectricity contribute to charge transfer in a similar way as described here. In the event of two metals, there might be no need to take flexoelectricity into consideration. Regarding non‐crystalline or semi‐crystalline materials, that is, polymers, the flexoelectricity is also supposed to affect the charge transfer in triboelectric measurement, nevertheless, it should be carefully analyzed because there could be plastic deformation under high forces and the mechanism of flexoelectricity in polymers is different from crystalline materials and could be dependent on various factors, such as motion and rotation of chains and cation sizes.^[^ ^66^, ^67^ ^]^

**Figure 4:**
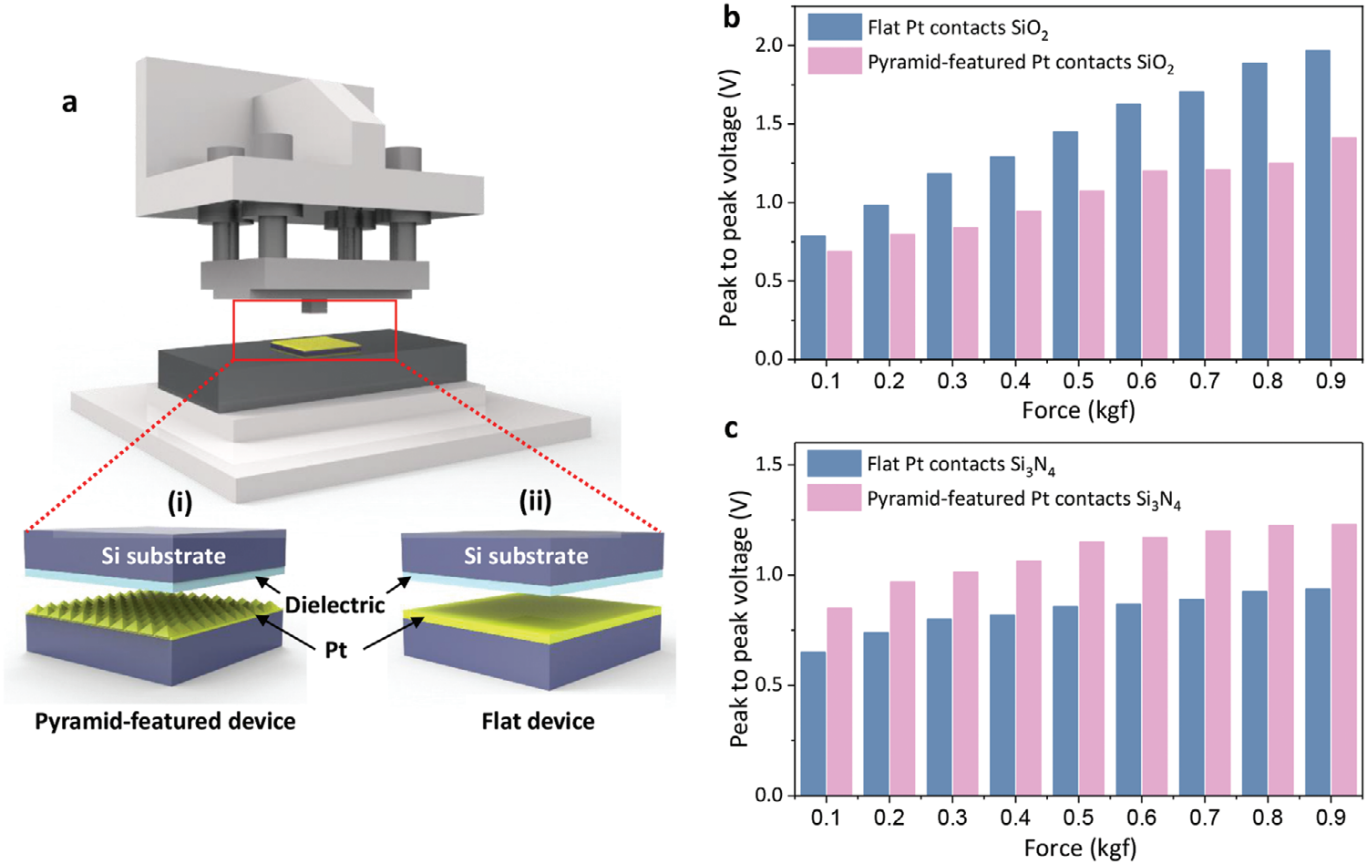
a) Schematic diagrams of triboelectric devices: i) pyramid‐featured Pt and ii) flat Pt. b) Peak‐to‐peak voltage as function of applied force (1 kgf = 9.8 n) in triboelectric devices of flat Pt and pyramid‐featured Pt contact b) SiO_2_ and c) Si_3_N_4_ thin films.

To further confirm the collaborative flexoelectric and triboelectric effects observed in the AFM, we fabricated triboelectric devices using a flat dielectric thin film and pyramid‐featured Pt thin film as the top and bottom layers, respectively, to simulate the conditions in the AFM measurements (**Figure** **4**a(i)). For comparison, a flat triboelectric device with flat layers of dielectric and Pt thin films (Figure 4a(ii)) was also fabricated and tested as a prototype, where only the triboelectric effect contributed to the output voltage. In both devices, the output voltage performance of the triboelectric devices was measured using a pushing tester (the upper image of Figure 4a). For comparison, two dielectric materials, SiO_2_ and Si_3_N_4_, were chosen for the triboelectric device measurements owing to the higher and lower work functions than Pt, which was examined based on KPFM measurements (Figures S8a and S8b, Supporting Information). In the case of pyramid‐featured Pt and SiO_2_, the triboelectric effect is suppressed by the flexoelectric effect owing to opposite charge transfer directions, which leads to a smaller peak‐to‐peak voltage in the pyramid‐featured device than in the flat one, as shown in Figure 4b. Furthermore, increasing the force causes a larger difference because of the increased flexoelectric effect. The detailed output voltages at different forces are shown in Figure S8c,d, Supporting Information. In contrast, the surface potential of Si_3_N_4_ is opposite that of SiO_2_, suggesting an opposite triboelectric charge transfer direction, which means that triboelectricity can be improved through the flexoelectricity. The results in Figure 4c distinctly indicate an enhanced triboelectric charging in the pyramid‐featured device, that is, the peak‐to‐peak voltage of the pyramid device is larger than that of the flat device (detailed output voltages are shown in Figure S8e,f, Supporting Information). Similarly, the difference between the pyramid‐featured and flat devices increases with increasing force. In both cases, the increasing contact area with force can be excluded as the main reason. The output voltage performance of the triboelectric device using TiO_2_ as dielectric material is also shown in Figure S8g, Supporting Information. The output voltage of nanopatterned device is smaller than that of flat one, similar with the SiO_2_ devices with slight difference, details can be found in Supporting Information. Consequently, the results of the flat and pyramid‐featured devices further verify the considerable contribution of concurrent flexoelectricity in triboelectric devices and suggest the selection of suitable material is important in a design of high efficiency nanopatterned devices.

## 3 Conclusion

In summary, we demonstrated the coupling between triboelectric and flexoelectric effects at the nanoscale, which could enhance charge transfer during triboelectric measurements when the charge transfers of both effects are in the same directions, or modulate the direction and amount of resulted charge transfer when they are in the opposite directions. In the AFM measurements, flexoelectricity is inevitably induced in the TiO_2_ film because of the strain gradient induced by the inhomogeneous force applied through the AFM tip. We showed that triboelectric and flexoelectric effects are fundamentally inseparable in nanoscale systems involving inhomogeneous stress/strain. The relative relationship between them could be modulated by normal force, which makes it possible to manipulate the amount as well as direction of the charge transfer in the case of opposite triboelectric and flexoelectric charging. In contrast, triboelectric charging can be improved through flexoelectricity when the charge transfer directions are the same. The mechanism can be applied to general scenarios involving an inhomogeneous strain at the nanoscale. The different performances of flat and pyramid‐featured triboelectric devices fabricated with different materials further support the proposed mechanism. This study can contribute to a fundamental understanding of the triboelectric effect where a dielectric material subjected to an inhomogeneous force is involved and could be further extended to increase the efficiency of the device performance in energy harvesting.

## 4 Experimental Section

### Material

A 100‐nm thick TiO_2_ thin film was synthesized using a radio frequency sputtering system (Ultech Co., Korea) on a Pt substrate. A commercial 300‐nm thick SiO_2_ thin film with a 525‐nm thick Si substrate (KCMC, Co.) was also used. The 100‐nm thick Si_3_N_4_ thin films were deposited on Si substrates using a 4‐in Si_3_N_4_ target (99.9%) through a radio frequency sputtering method.

### AFM Measurements

AFM measurements were performed using a commercially available AFM (NX10, Park Systems). A conductive diamond‐coated AFM tip (CDT‐FMR, NanoSensors, thermal tune‐calibrated spring constant *k* of ≈7.2 N m^−1^) and Pt‐coated AFM tip (Multi75E‐G (BudgetSensors, with a thermal tune‐calibrated spring constant *k* of ≈4.2 N m^−1^) were used in the experiments. The surface potential was obtained using amplitude‐modulated KPFM mode by applying a 2 V AC voltage at 17 kHz and a DC feedback voltage to the AFM tip, a scan rate of 0.5 Hz was used. The effective work function of the tip was calibrated using highly ordered pyrolytic graphite (HOPG, Park Systems). The *I*–*V* curves were measured using a function generator (PXIe‐1062Q, National Instruments) controlled through LabVIEW/MATLAB‐based software. All experiments were conducted in air with a relative humidity of ≈27% and a temperature of ≈30 °C. A silicon grating with trapezoidal steps (TGF11) for a flat‐wedge friction force calibration was purchased from MikroMasch.

### Fabrication of Triboelectric Devices

A 2 × 2 cm^2^ pyramid‐featured Si substrate was fabricated using KOH etching at 60 °C for 30 min. A 100‐nm thick Pt film was deposited on flat and pyramid‐featured Si substrates using an electron beam evaporator as the bottom layer. The top layer size was controlled at 1 × 1 cm^2^ to ensure the same contact area.

## Conflict of Interest

The authors declare no conflict of interest.

## Supporting information

Supporting Information

## Data Availability

Research data are not shared.
