# Supplementary material for: Mixed Triboelectric and Flexoelectric Charge Transfer at the Nanoscale
Source: Adv Sci (Weinh). 2021 Aug 13;8(20):2101793. doi: 10.1002/advs.202101793 (PMC8529448; doi:10.1002/advs.202101793)
Supplement: Supplementary file 1 — Supporting Information [file ADVS-8-2101793-s001.pdf]

WILEY-VCH

© 2021 Wiley-VCH GmbH

ADVANCED SCIENCE
Open Access

Supporting Information

for *Adv. Sci.*, DOI: 10.1002/advs.202101793

Mixed triboelectric and flexoelectric charge transfer at the nanoscale

Huimin Qiao, Pin Zhao, Owoong Kwon, Ahrum Sohn, Fangping Zhuo, Dong-Min Lee, Changhyo Sun, Daehee Seol, Daesu Lee, Sang-Woo Kim, and Yunseok Kim**

Supplementary Information

Mixed triboelectric and flexoelectric charge transfer at the nanoscale

Huimin Qiao, Pin Zhao, Owoong Kwon, Ahrum Sohn, Fangping Zhuo, Dong-Min Lee, Changhyo Sun, Daehee Seol, Daesu Lee, Sang-Woo Kim, and Yunseok Kim**

Dr. H. Qiao, P. Zhao, O. Kwon, Dr. A. Sohn, D.-M. Lee, C. Sun, Dr. D. Seol, Prof. S.-W. Kim, Prof. Y. Kim
School of Advanced Materials and Engineering, Sungkyunkwan University (SKKU), Suwon 16419, Republic of Korea
E-mail: kimsw1@skku.edu (S.-W.K.), yunseokkim@skku.edu (Y.K.)

Dr. H. Qiao, O. Kwon, C. Sun, Prof. Y. Kim
Research Center for Advanced Materials Technology, Sungkyunkwan University (SKKU), Suwon 16419, Republic of Korea

Dr. F. Zhuo
Department of Materials and Earth Sciences, Technical University of Darmstadt, Darmstadt, 64287, Germany

Prof. D. Lee
Department of Physics, Pohang University of Science & Technology (POSTECH), Pohang, 37673, Republic of Korea

Prof. S.-W. Kim
SKKU Advanced Institute of Nanotechnology (SAINT) and Samsung Advanced Institute for Health Sciences & Technology (SAIHST), Sungkyunkwan University (SKKU), Suwon 16419, Republic of Korea

1. Work function calibration

Based on the mechanism of KPFM, the contact potential difference (V_{CPD}) between the conducting AFM tip and the sample can be expressed as follows:

$$V_{CPD} = \frac{\varphi_{tip} - \varphi_{sample}}{-e}, \tag{1}$$

where φ_{tip} and $\varphi_{\mathrm{sample}}$ are the work functions of the tip and sample respectively, and e is the electronic charge.[1] Therefore, using a calibration sample, *e.g.*, HOPG, with the known work function, we can easily calibrate the work function of the sample from Equation (1). The surface potential image measured on HOPG by the tip CDT-FMR used in the experiments is shown in Figure S1a, whereas the surface potential image of the TiO_2 thin film with the same tip is shown in Figure S1b. Note that we conducted the measurements at several regions on the sample surface and obtained an average value for the calculation. The effective work function of the tip and sample can then be calculated as follows:

$$\varphi_{\mathrm{tip}} = \varphi_{\mathrm{HOPG}} - eV_{\mathrm{CPD}} = 4.65\mathrm{eV} - 0.13\mathrm{eV} = 4.52\ \mathrm{eV}, \tag{2}$$

$$\varphi_{\mathrm{sample}} = \varphi_{\mathrm{tip}} + eV_{\mathrm{CPD}} = 4.52\mathrm{eV} + 0.28\mathrm{eV} = 4.8\ \mathrm{eV}. \tag{3}$$

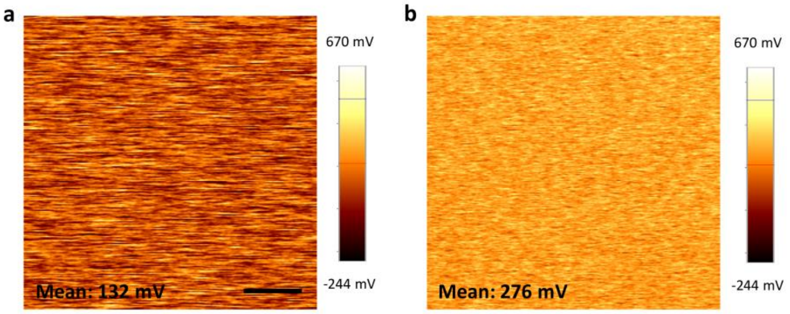

Figure S1. Surface potential images on (a) HOPG and (b) TiO_2 thin film. Scale bar is 1 μm.

2. Friction force calibration

The improved Wedged-Flat method was used for a friction force calibration to reduce the errors of adhesion force measurement, static coupling between normal and friction signals, and optical interference.[2] The friction loops were obtained on the Si grating under seven normal forces ranging from 200 to 800 nN. For each normal force, 64 friction loops were collected to obtain an averaged loop, as shown in Figure S2a. The distance of the friction loop measurement was set to 10 μm to include the positive and negative slopes and the flat portion of the grating. A scan rate of 0.5 Hz was set to ensure good contact between the tip and grating surface. From the friction loop, we were able to obtain the value of the half-width (W_0) of the friction loop on the slope, and the relative friction loop offset ($\Delta_0(\theta)-\Delta_0(0)$) by subtracting the friction loop offset on the flat surface from that on the sloped surface. The friction coefficient μ and friction force sensitivity $OLFS_F$ can be determined by solving the following equations:[2]

$$S_W = \frac{\mu}{cos^2\theta - \mu^2 sin^2\theta} OLFS_F, \quad (4)$$

$$S_\Delta = \frac{(\mu^2+1) sin\theta cos\theta}{cos^2\theta - \mu^2 sin^2\theta} OLFS_F, \quad (5)$$

where S_W and S_Δ are the slopes of the linear fitting curves for W_0 and $\Delta_0(\theta)-\Delta_0(0)$ versus the normal force, respectively. The values of θ for the positive and negative slopes were 54.74° and −54.74°, respectively. The experimental and fitting results are shown in Figure S2b (b), the calibration results are summarized in Table S1.

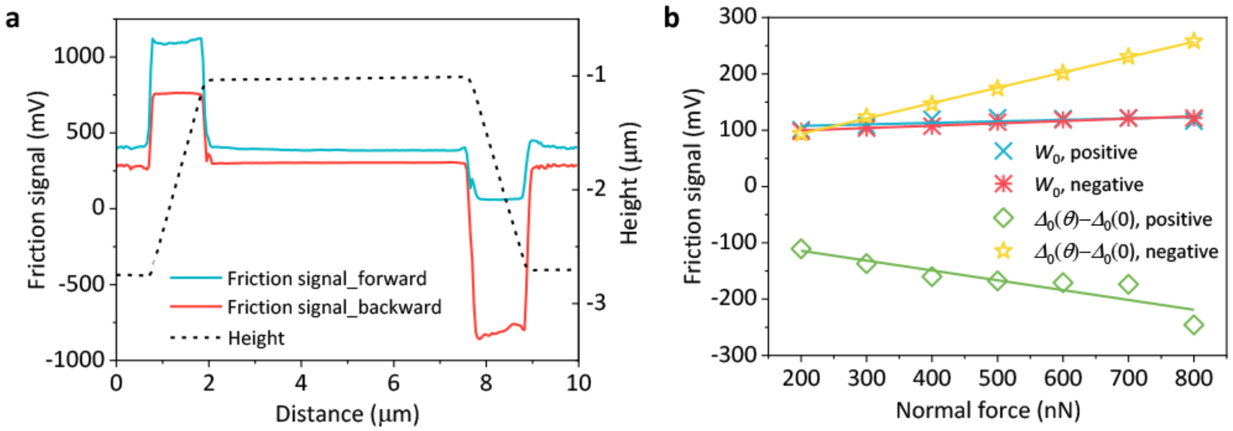

Figure S2. (a) Example of friction loop showing friction displacement dependent friction voltage signal (normal force of 300 nN). (b) Plots of W_0 and $\Delta_0(\theta)$-$\Delta_0(0)$ as function of normal force; solid lines are linear fitting results.

Table S1. Summary of friction force calibration results.

Slope	S_W (mV/nN)	S_Δ (mV/nN)	μ	$OLFS_F$(V/μN)	Correction factor (μN/V)
54.74°	0.19	1.5574	0.0577	1.0901	0.9132
−54.74°	1.1503	−1.9513	0.3035	1.0305	0.966

3. Calculation of strain and strain gradient distribution and flexoelectric surface potential

The Hertzian model[3] is widely used for single non-adhesive asperity contact. Herein, we employed this classical model to describe the deformations of a rigid sphere on an elastic flat half-space and derive analytical expressions for the strain and strain gradients. In cylindrical coordinates, the stress distributions are given by the following:[3]

$$\frac{\sigma_{rr}}{p_m} = \frac{3}{2}\left\{\frac{1-2\nu}{3}\frac{a^2}{r^2}\left(1-\left(\frac{z}{\sqrt{u}}\right)^3\right)+\left(\frac{z}{\sqrt{u}}\right)^3\frac{a^2u}{u^2+a^2z^2}\right.$$
$$\left.+\frac{z}{\sqrt{u}}\left(u\frac{1-\nu}{a^2+u}+\frac{(1+\nu)\sqrt{u}}{a}tan^{-1}\left(\frac{a}{\sqrt{u}}\right)-2\right)\right\} \quad (6)$$

,

$$\frac{\sigma_{\theta\theta}}{p_m} = -\frac{3}{2}\left\{\frac{1-2\nu}{3}\frac{a^2}{r^2}\left(1-\left(\frac{z}{\sqrt{u}}\right)^3\right)+\frac{z}{\sqrt{u}}\left(2\nu+u\frac{1-\nu}{a^2+u}-\frac{(1+\nu)\sqrt{u}}{a}tan^{-1}\left(\frac{a}{\sqrt{u}}\right)\right)\right\} \quad (7)$$

,

$$\frac{\sigma_{zz}}{p_m} = -\frac{3}{2}\left(\frac{z}{\sqrt{u}}\right)^3\frac{a^2u}{u^2+a^2z^2}, \quad (8)$$

$$\frac{\sigma_{rz}}{p_m} = -\frac{3}{2}\frac{rz^2}{u^2+a^2z^2}\frac{a\sqrt{u}}{a^2+u}, \quad (9)$$

where r and z are cylindrical coordinates, v is the Poisson's ratio, and a is the deformation radius. We then obtain the following equations:

$$\mathrm{a} = \left(\frac{3}{4}\frac{FR}{Y}(1-\nu^2)\right)^{\frac{1}{3}}, \quad (10)$$

$$p_m = \frac{F}{\pi a^2}, \quad (11)$$

$$u = \frac{1}{2}\left(r^2+z^2-a^2+\sqrt{(r^2+z^2-a^2)^2+4a^2z^2}\right), \quad (12)$$

where F is the applied force, Y is the effective Young's modulus, and R is the indenter radius. In addition, Y can be calculated through the following:

$$\frac{1}{Y} = \frac{1-\nu^2}{Y_s} + \frac{1-\nu_{tip}^2}{Y_{tip}}, \tag{13}$$

where ν_{tip} (0.20) and Y_{tip} (1050 GPa) are the Poisson's ratio and Young's modulus of the diamond-coated AFM tip, respectively. In addition, Y_S (120 GPa) is the Young's modulus of the TiO_2 sample. These stresses are related to strain using the isotropic Hooke's law, as given in the following equations:[3]

$$\varepsilon_{rr} = \frac{\sigma_{rr}-\nu(\sigma_{\theta\theta}+\sigma_{zz})}{Y}, \tag{14}$$

$$\varepsilon_{\theta\theta} = \frac{\sigma_{\theta\theta}-\nu(\sigma_{rr}+\sigma_{zz})}{Y}, \tag{15}$$

$$\varepsilon_{zz} = \frac{\sigma_{zz}-\nu(\sigma_{\theta\theta}+\sigma_{zz})}{Y}, \tag{16}$$

$$\varepsilon_{rz} = \frac{2(1+\nu)}{Y}\sigma_{rz}. \tag{17}$$

Owing to the radial symmetry, no θ dependence formulas were included. The cylindrical strains can then be transformed into Cartesian strains using the following matrix:

$$\mathrm{T} = \begin{bmatrix} cos(\theta) & -sin(\theta) & 0 \\ \sin(\theta) & \cos(\theta) & 0 \\ 0 & 0 & 1 \end{bmatrix}. \tag{18}$$

The normal component of the flexoelectric coupling field in an isotropic dielectric material is given by the following:[4]

$$E_z = f\left(3\varepsilon_{zzz} + \varepsilon_{zxx} + \varepsilon_{xzx} + \varepsilon_{xxz} + \varepsilon_{zyy} + \varepsilon_{yzy} + \varepsilon_{yyz}\right). \tag{19}$$

In these expressions, f is the flexoelectric coefficient normalized by the dielectric constant, that is, flexocoupling voltage, and the symmetry-allowed strain gradients (ε_{jkl}) are denoted explicitly ($\varepsilon_{jkl} = \partial\varepsilon_{jk}/\partial x_l$). For the TiO_2 case, the flexocoupling voltage is 1.3 V.[5] The flexoelectric field in the bulk of the deformed body will generate a surface potential difference,

which can be calculated from the normal component of the flexoelectric field through the following:

$$\mathrm{V}(x, y) = \int_0^\infty E_z(x, y, z) dz. \tag{20}$$

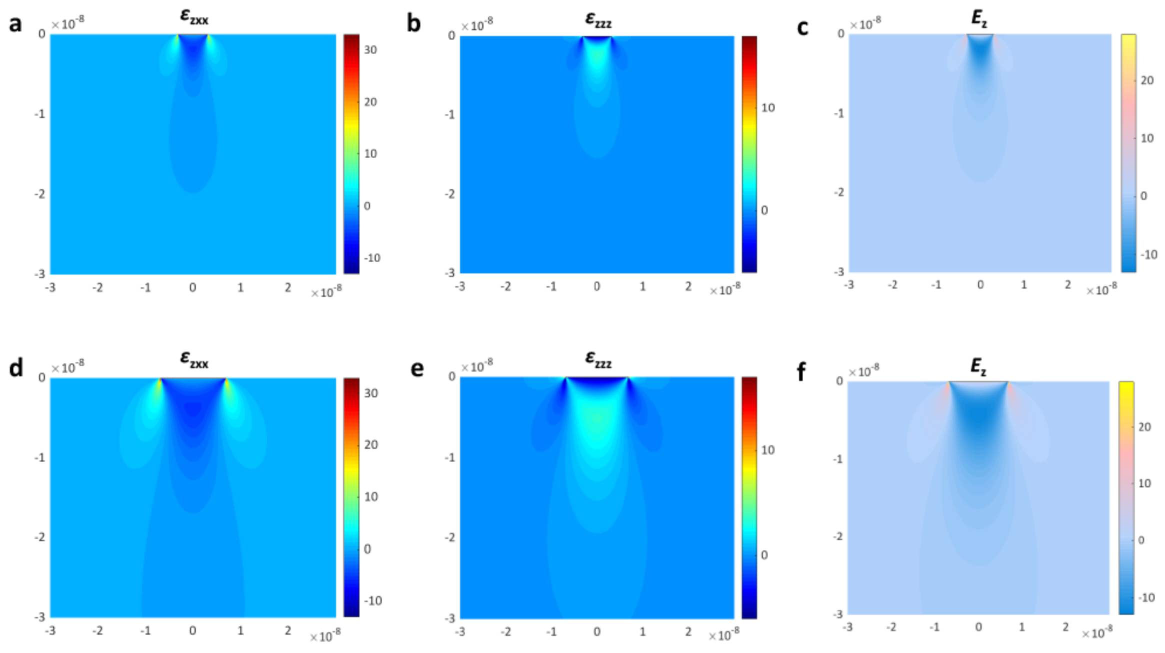

Figure S3. (a–e) Distribution of strain gradient of (a, d) ε_{zxx} and (b, e**)** ε_{zzz}. (c, f**)** Flexoelectric coupling field E_z. Normal force: (a–c) 50 nN and (d–f) 400nN.

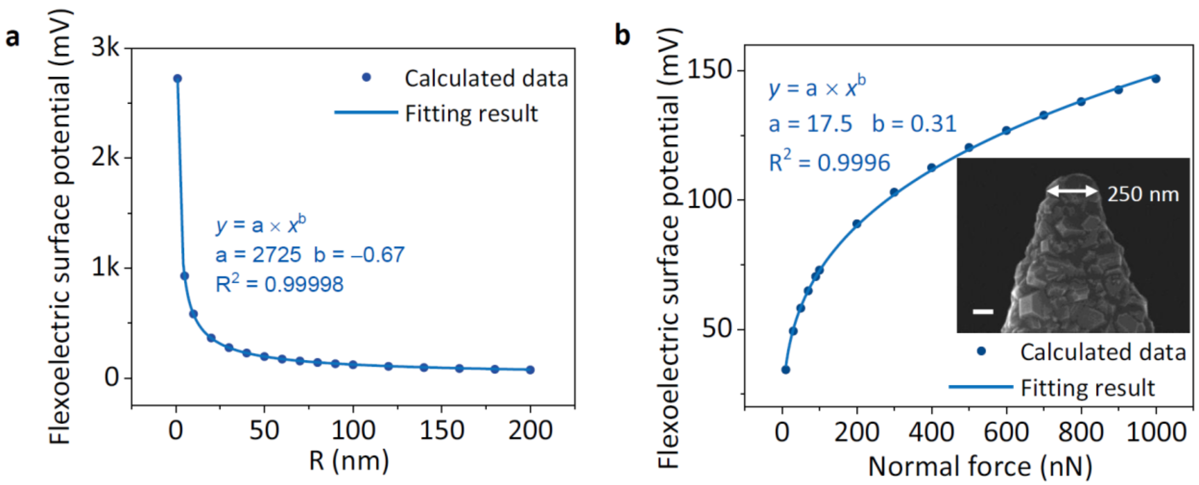

Figure S4. Calculated (a) tip-radius dependent and (b) normal-force dependent flexoelectric surface potentials. Normal force of 500 nN and tip radius of 100 nm were used for each calculation, respectively. Inset of (b) is SEM image of tip apex. Scale bar is 100 nm. Based on SEM image of (b), tip radius of 100 nm was used for calculation of flexoelectric surface potential at different normal forces.

The tip radius- and normal force-dependent flexoelectric surface potentials are shown in Figs. S4a and S4b. The results indicate that the flexoelectric surface potential is sensitive to both the tip radius and normal force, and it decreases with the tip radius and increases with the normal force. The fits indicate the exponential dependence of the flexoelectric surface potential on both the tip radius and normal force.[4] The flexoelectric surface potential decreases dramatically from a large value with an increasing tip radius when it is below 50 nm, and becomes slow at above 50 nm. Therefore, the flexoelectric surface potential is not very sensitive to the tip radius within the range of ~100 nm in this study.

4. Results with different sample and tip

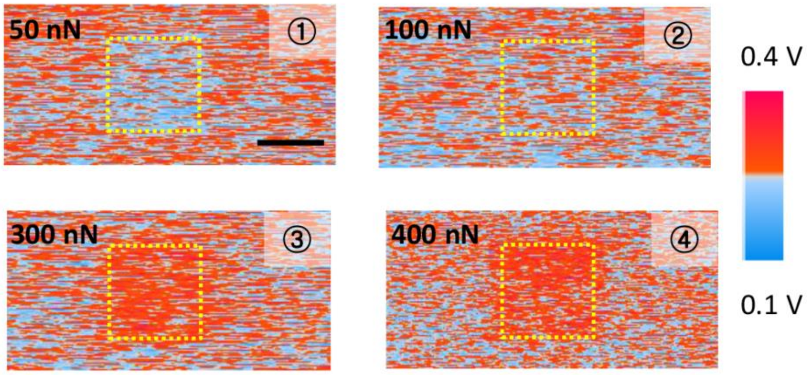

Figure S5. Surface potential images after rubbing with different normal forces on TiO_2 thin film by Pt-coated tip (Multi75E-G). Numbers indicate measurement sequence. Scale bar is 3 μm.

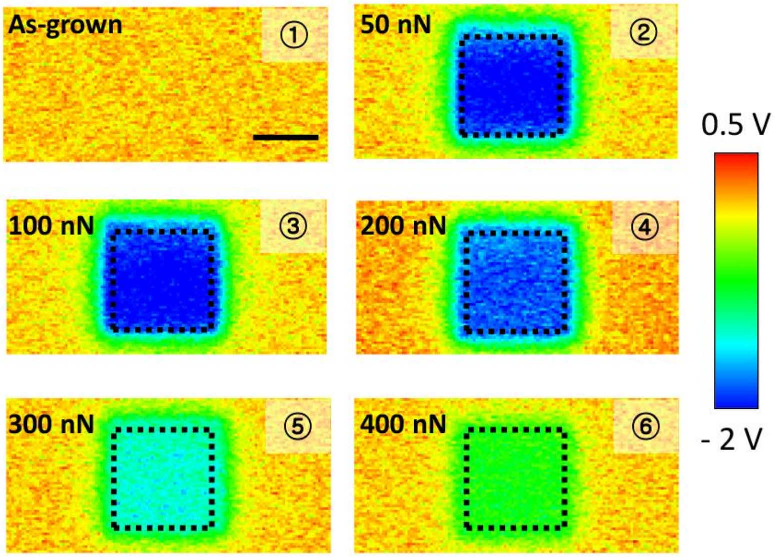

Figure S6. Surface potential images after rubbing with different normal forces on SiO_2 thin film by conductive diamond-coated tip (CDT-FMR). Numbers indicate measurement sequence. Scale bar is 3 μm.

The charge transfer behavior induced using a Pt-coated AFM tip (Multi75E-G) on the TiO_2 thin film is shown in Figure S5. Likewise, the center area is negatively charged after rubbing with a low normal force, and gradually becomes positively charged when the normal force is increased. Figure S6 shows the charge transfer behavior at normal force from 50 to 400 nN on the SiO_2 thin film. Similar to the charge transfer on the TiO_2 thin film, the triboelectric charge on SiO_2 decreases with increasing normal force. In this case, it is notable that the surface potential decreases by almost −1.5 V after rubbing with a normal force of 50 nN, which is much larger than that in TiO_2. As mentioned in the main text, because the triboelectric charge is strongly affected by the surface state,[6, 7] the influence of the surface state on the triboelectric charge in SiO_2 might be more notable than that in the TiO_2 thin film. The results obtained in Figs. S5 and S6 suggest that the phenomenon described in the main text is not unique to a specific sample or tip.

5. Local I-V curves at varying normal force

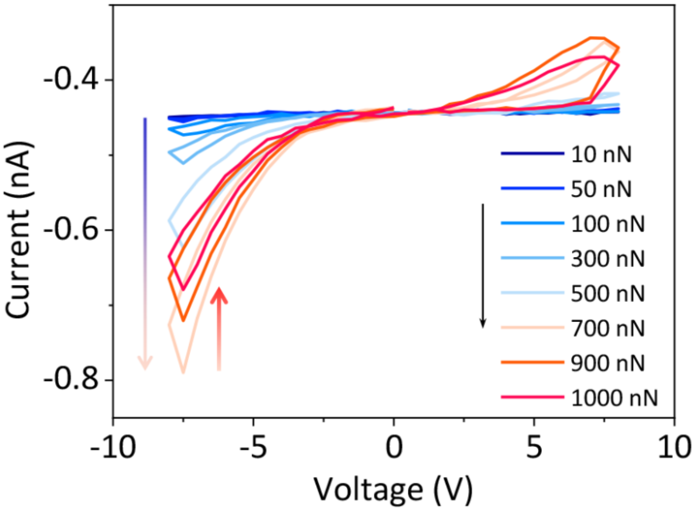

Figure S7. Local I-V curves of TiO_2 thin film measured at different normal force. Black arrow shows measurement sequence.

The normal force-dependent local I-V curves of the TiO_2 thin film are shown in Figure S7, based on a Pt-coated cantilever used for the measurements. All loops show an asymmetric shape with a negative forward bias, which has been reported as a consequence of the Schottky-like barrier at the interface,[8] which is mainly related to the work function difference between the tip and sample in this case. In general, the current flow monotonically increases with an increase in the normal force because of the increasing contact area.[9] In Figure S7, however, it is clearly observed that the negative current first increases until 700 nN and then decreases as the normal force increases. We can attribute the abnormal variation of current above 700 nN to the increasing flexoelectricity. The Fermi level of the sample can be affected by the flexoelectric field when the normal force is sufficiently high, which could weaken the triboelectricity in question.[10] Therefore, the current flow can drop when the flexoelectricity is significant at a high normal force. It is notable that the critical normal force (700 nN) is much higher than in the

scanning experiment, as displayed in Figure 1, one of the possible reasons for which is the larger influence of the normal force on the contact area in the I-V curve measurement (one point) than in scanning mode ($3 \times 3\ \mu m^2$); therefore, the critical normal force differs significantly in these two cases. By contrast, the tip used in this experiment was a Multi75E-G, which is not as stiff as a CDT-FMR; therefore, the wear of the tip apex can also contribute to the difference.

6. Triboelectric performance in different systems

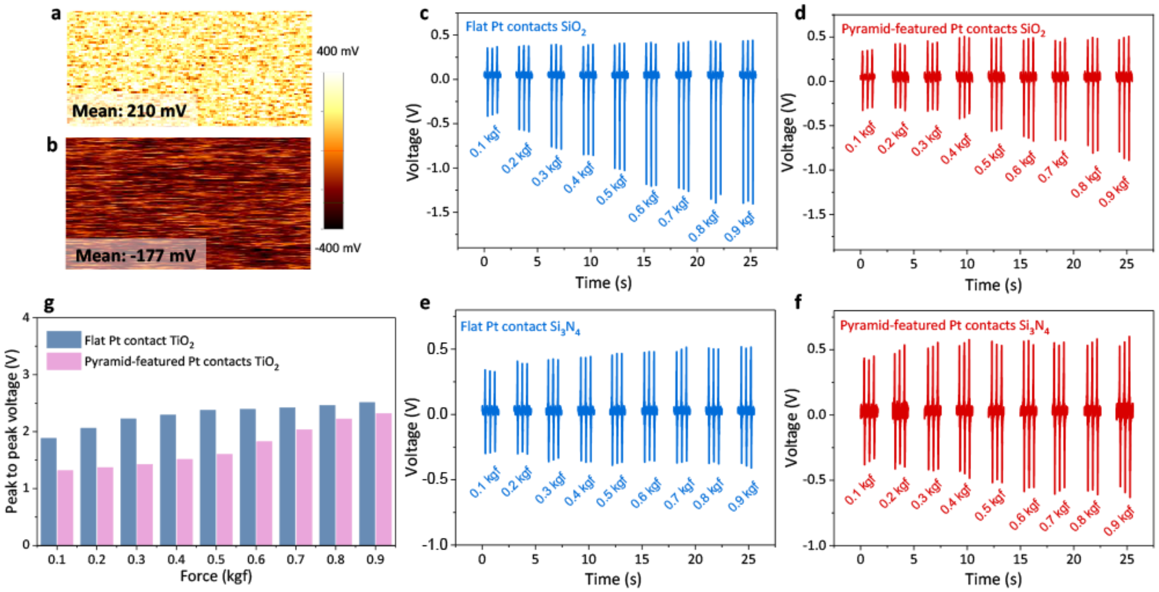

Figure S8. (a, b) Surface potential images on (a) SiO_2 and (b) Si_3N_4 thin film measured by Pt-coated cantilever. Output voltage details for (c) flat Pt contacts SiO_2, (d) pyramid-featured Pt contacts SiO_2, (e) flat Pt contacts Si_3N_4, and (f) pyramid-featured Pt contacts Si_3N_4. (g) Peak to peak voltage as a function of applied force in triboelectric devices of flat Pt and pyramid-featured Pt contact TiO_2.

In triboelectric devices fabricated by TiO_2 thin film as dielectric material, the output voltage difference between flat and nanopatterned devices shows a similar tendency with that in SiO_2 devices at forces below 0.3 kgf, while it is slightly different with SiO_2 at high forces. We notice that the flat device reaches a saturated state at high forces, while the SiO_2 flat device does not show saturation in the applied force range. The different saturation state could be a reason for the slight difference between devices made by different materials.

References

[1] R. Shikler, T. Meoded, N. Fried, B. Mishori, Y. Rosenwaks, *J. Appl. Phys.* **1999**, *86*, 107.

[2] B. C. T. Khac, K. H. Chung, *Ultramicroscopy* **2016**, *161*, 41.

[3] A. C. Fischer-Cripps, *Introduction to contact mechanics*, Springer-Verlag, New York, **2000**.

[4] C. A. Mizzi, A. Y. W. Lin, L. D. Marks, *Phys. Rev. Lett.* **2019**, *123*, 116103.

[5] J. Narvaez, F. Vasquez-Sancho, G. Catalan, *Nature* **2016**, *538*, 219.

[6] K. E. Byun, Y. Cho, M. Seol, S. Kim, S.-W. Kim, H.-J. Shin, S. Park, S. Hwang, *ACS Appl. Mater. Interfaces* **2016**, *8*, 18519.

[7] D. R. Islamov, V. A. Gritsenko, T. V. Perevalov, O. M. Orlov, G. Y. Krasnikov, *Appl. Phys. Lett.* **2016**, *109*, 052901.

[8] W. H. Lu, L.-M. Wong, S. J. Wang, K. Y. Zeng, *J. Materiomics* **2018**, *4*, 228.

[9] L. L. Shu, S. M. Ke, L. F. Fei, W. B. Huang, Z. G. Wang, J. H. Gong, X. N. Jiang, L. Wang, F. Li, S. J. Lei, Z. G. Rao, Y. B. Zhou, R. K. Zheng, X. Yao, Y. Wang, M. Stengel, G. Catalan, *Nat. Mater.* **2020**, *19*, 605.

[10] J. Quereda, J. J. Palacios, N. Agräit, A. Castellanos-Gomez, G. Rubio-Bollinger, *2D Mater.* **2017**, *4*, 021006.
